# Expression deregulation of *mir31* and *CXCL12* in two types of oral precancers and cancer: importance in progression of precancer and cancer

**DOI:** 10.1038/srep32735

**Published:** 2016-09-06

**Authors:** Esita Chattopadhyay, Richa Singh, Anindita Ray, Roshni Roy, Navonil De Sarkar, Ranjan Rashmi Paul, Mousumi Pal, Ritesh Aich, Bidyut Roy

**Affiliations:** 1 Human Genetics Unit, Indian Statistical Institute, Kolkata, India; 2 Department of Oral Pathology, Guru Nanak Institute of Dental Science & Research, 157/F Nilganj Road, Kolkata, 700114, India; 3 Department of Oral Medicine, Dr. R Ahmed Dental College and Hospital, 114- AJC Bose Road, Kolkata 700014, India

## Abstract

Oral cancer generally progresses from precancerous lesions such as leukoplakia (LK), lichen planus (LP) and oral submucous fibrosis (OSMF). Since few of these precancers progress to cancers; it is worth to identify biological molecules that may play important roles in progression. Here, expression deregulation of 7 miRNAs (mir204, mir31, mir31*, mir133a, mir7, mir206 and *mir1293*) and their possible target genes in 23 cancers, 18 LK, 12 LP, 23 OSMF tissues compared to 20 healthy tissues was determined by qPCR method. Expression of mir7, mir31, mir31* and *mir1293* was upregulated and that of *mir133a, mir204* and *mir206* was downregulated in cancer. Expression of most of these miRNAs was also upregulated in LK and LP tissues but not in OSMF. Expression deregulation of some of the target genes was also determined in cancer, LK and LP tissues. Significant upregulation of *mir31* and downregulation of its target gene, *CXCL12*, in cancer, LK and LP tissues suggest their importance in progression of precancer to cancer. Expression upregulation of *mir31* was also validated using *GEO* data sets. Although sample size is low, novelty of this work lies in studying expression deregulation of miRNAs and target genes in oral cancer and three types of precancerous lesions.

Among all the non-coding RNAs, the most studied category is microRNA (miRNA) which is 20–22 nucleotide single stranded RNAs known for gene silencing by mRNA degradation or inhibition of translation. Each miRNA can regulate expression of many target genes and expression of each target gene may also be regulated by multiple miRNAs. By this miRNA-mRNA interaction, miRNAs take part in fundamental cellular processes like cell proliferation, differentiation, apoptosis, stress response etc.

Primarily, miRNAs were frequently found to be located in fragile genomic sites and genomic regions which were found to be associated with cancer risk^1^. This led to the hypothesis that miRNAs might have some role in carcinogenesis. More recent studies have proved the definite signature of miRNAs in initiation and progression of cancer. Based on their expressions and functions in cancer tissue, miRNAs are also classified as tumor suppressor and oncogenic miRNAs. Since expression profiles of miRNAs are very different from one cancer type to another, they bring the opportunity of using them as cancer specific biomarkers^2^.

Some of the pre-cancerous lesions progress to cancer, in spite of report of tumorigenesis from normal tissues. In expression profiling study of miRNAs in oral cancer tissue, we reported that expression of 7 miRNAs was found to be significantly deregulated in cancer with respect to their adjacent normal tissues^3^. The purpose of this study was to compare expression of those seven miRNAs and some of their target genes in oral cancer and pre-cancer compared to normal tissues from different set of healthy individuals.

## Results

### miRNA expression in cancer tissues

Expressions of 7 miRNAs in cancer and precancer tissues were compared with respect to those in normal tissues from healthy individuals. At least 2 fold expression change in disease tissue (up or down-regulation) compared to the normal was considered to be the threshold value for calling it deregulation in expression since *C*_*T*_ values of duplicate of a sample vary by less than 1 *C*_*T*_ value i.e. less than 2 fold change. Expression of all 7 miRNAs were found to be significantly deregulated in cancer samples, 4 of them were up-regulated (hsa-miR-7, hsa-miR-31, hsa-miR-31* and hsa-miR-1293) while expression of remaining 3 miRNAs were down-regulated (*hsa-miR-133a, hsa-miR-204, hsa-miR-206*) as was observed in a previous report in which adjacent control was used to compare the expression deregulation^3^ (Table 1). But it is important to note that both the disease and normal tissues were collected from the same cancer patients in the previous report^3^ whereas cancer and normal tissues were taken from two different set of individuals in this study (Table 1 and Supplementary Figure S1). The direction of expression (up or down) deregulation was found to be perfectly matched for each miRNA, though the magnitude of expression (i.e. fold changes) showed differences between this study and our previous study (Table 1).

### miRNA expression in three types of pre-cancer tissues

Except *hsa-miR-204,* expression of remaining 6 miRNAs, were found to be significantly up-regulated in leukoplakia. Similarly, except *hsa-miR-133a* and *hsa-miR-206*, expression of remaining 5 miRNAs was significantly up-regulated in lichen planus tissues (Table 2 and Supplementary Figure S1). In OSMF samples, expression of *hsa-miR-31* and *hsa-miR-204* were significantly up-and down regulated, respectively. Expression of hsa-miR-7, hsa-miR-31, hsa-miR-31* and *hsa-miR-1293* was significantly up-regulated in cancer, leukoplakia and lichen planus samples and that of *hsa-miR-204* was significantly down-regulated in cancer and OSMF samples. *Hsa-miR-31* is the only miRNA which was significantly up-regulated in cancer and all precancer tissues (Table 2).

### Expression of target genes in cancer and pre-cancer tissues

For *miR-204*, five target genes (*MMP9, PLAUR, SERPINE1, SNAI2* and *COL5A3*); for *miR-31,* three target genes (*DMD, CXCL12* and *WASF3*); for miR-31*, one target gene (*KIAA1737);* for *miR-7*, one target gene (*TGM2);* for *miR-133a*, three target genes (*H2AFX, RUNX3* and *MTHFD1L* and for *miR-206*. one target gene (*FYB)* were selected for expression study (Table 3, Supplementary Figures S2–S5). Expression of *miR-204* was significantly down-regulated in cancer and OSMF tissues, while expression of its three target genes (*MMP9, PLAUR* and *SERPINE1*) showed significant up-regulation (Table 4 and Supplementary Figure S2) in cancer only. In contrast, expression of *miR-31* was significantly up-regulated in cancer, LK, LP and OSMF tissues while expression of one target, *CXCL12,* was significantly down-regulated in cancer, LK and LP tissues. In LK and LP samples, significant downregulation in expression was noticed for *WASF3* which is a possible target of *mir31* (Table 4, Supplementary Figure S3). OSMF samples were sub-grouped on the basis of presence or absence of dysplasia. Expression of miRNAs and target genes in these two sub groups of OSMF were compared but no statistically significant difference in expression of any miRNAs or target genes was found (data not shown). From our whole transcriptome data from cancer tissues (unpublished data) it was found that expression of three target genes of *miR-133a* and one target gene of *miR-206* was significantly up-regulated (Table 4).

### Validation miRNA expression using GEO data sets

Upon analysis, *GSE33299* data set showed upregulation of expression of *miR-31* in both leukoplakia and leukoplakia-transformed cancer with respect to normal tissue but expression was more in leukoplakia-transformed cancer tissues (data not shown). Similarly, another data set, *GSE62809* revealed that *miR-31* to be one of the highly expressed miRNAs in both progressive and non-progressive leukoplakia groups but its expression was higher in progressive leukoplakia group (data not shown). Expression of 7 miRNAs (mir204, mir31, mir31*, mir133a, mir7, mir206 and *mir1293)* in our study showed similar patterns of expression deregulation like *GSE33299* data set except *miR-7* and *miR-206* in leukoplakia and leukoplakia-transformed cancer tissues, respectively. Apart from *mir31, GSE62809* data set also showed similar trend in expression deregulation of miR-31* and *miR-204* but remaining miRNAs were not considered for expression analysis due to low read count.

## Discussion

Expression of seven miRNAs and fourteen target genes were studied in oral cancer, 3 types of pre-cancer and normal tissues from 5 five different sets of individuals. It is observed that miRNA expressions in oral cancer tissues were consistent with the result of the previous study^3^ on a different set of cancer and adjacent control tissues. It is noteworthy to mention that the control tissues were taken from a different set of healthy individuals in this study whereas it was adjacent normal tissue in previous study. But expression deregulation of miRNAs in cancer tissues is in same direction (i.e. up- or down-regulation) when compared with control tissues collected from either adjacent site of cancer tissue or healthy individuals (Table 1). In case of leukoplakia, expression deregulation of only *mir-31* and mir-31* was in same direction when compared with control tissues from either adjacent site^3^ or different healthy individuals (Table 2). In Lichen planus tissues, expression of none of these miRNAs was deregulated when compared to adjacent controls^3^ but most of them have deregulated expression when compared to control tissues taken from different healthy individuals (Table 2). In this study, most of the seven miRNAs was found to be up-regulated in LK and LP with respect to normal tissues collected from healthy individuals (Table 2). But in OSMF tissue, expression of *miR-31* and *miR-204* was significantly deregulated like cancer tissue (i.e. *miR-31* up-regulated and *miR-204* down-regulated). So, we observed that expression of *miR-31* was upregulated in cancer and all precancer tissues (Table 4) and similar result was also reported in other studies on different cancers and oral precancer tissues^4^,^5^,^6^,^7^. GEO datasets (*GSE33299, GSE62809*) was used for expression validation and it revealed that *miR-31* expression is up-regulated in oral leukoplakia and leukoplakia-transformed cancer samples, thus, suggesting importance of *miR-31* upregulation in progression of leukoplakia to cancer^8^. *Mir-31* is capable to promote tumor growth and has oncogenic potential in head and neck and other cancers^9^,^10^,^11^. In contrast, it is also reported to be down-regulated in cancer and prevent metastasis in cancer cells^12^. Like *miR-31,* expression of miR-31* is also up-regulated in cancer and two pre-cancer tissues in this study like a report in another study^13^, but its expression was found to be significantly associated with progression free survival of metastatic colorectal cancer^14^. *MiR-7* is a known OncomiR and its expression was up-regulated in cancer, leukoplakia and lichen planus tissues in this study, like in different cancers from other report^15^. Expression of *miR-133a, miR-204* and *miR-206* was down-regulated in cancer tissues but up-regulated in precancer tissues in this study. Their expressions are also found to be down-regulated in head and neck and other cancers and considered as tumor-suppressors^16^,^17^,^18^. Restoration of expression of these miRNAs causes significant inhibition of cell proliferation, migration, invasion and tumorigenesis as well as significant increase in apoptosis^16^,^18^,^19^,^20^,^21^,^22^. Only few reports are available regarding expression of *miR-1293* in cancer^3^ but expression was found to be up-regulated in cancer, leukoplakia and lichen planus tissues in this study (Table 2).

In this study, expression of miRNAs and target genes was significantly deregulated in both cancer and precancer tissues (Table 4). Expression of three target genes *(H2AFX, RUNX3* and *MTHFD1L)* of *miR-133a* and one target gene (*FYB*) of *miR-206* was upregulated while expression of these two miRNAs (*miR-133a* and *miR-206)* were significantly downregulated in cancer tissues (Table 4). *H2AFX* is one of the histone family member gene which are involved in DNA double strand break repair pathway^23^. Although methylation, copy number alterations, deletion and single nucleotide polymorphisms in *H2AFX* are well-linked with different types of cancer, expression deregulation of the gene is also reported in a few studies^24^,^25^,^26^,^27^. *RUNX3* is a transcription factor that functions as a tumor-suppressor in bladder, colorectal, hepatocellular, lung and gastric cancer^28^,^29^. But in head and neck cancer it plays an oncogenic role. Upregulation of *RUNX3* in head and neck cancer leads to increased cell proliferation and reduced apoptosis^30^. *MTHFD1L* is an enzyme that works in the mitochondrial pathway of glycine biosynthesis. Higher expression of glycine pathway genes have been reported to induce rapid proliferation in cancer cells as well as associated with greater mortality of cancer patients^31^. *FYB* (also known as *ADAP* and *SLAP-130*) participates in T-cell receptor mediated actin cytoskeletal rearrangement with activation of integrins function, T cell migration and production of cytokines^32^,^33^,^34^. Knockout of *FYB* gives protection against tumor formation and metastasis in mice^35^. So, expression deregulation of *FYB* might impair the immune system of cancer cells. Significant upregulation of expression of 3 target genes (*MMP9, PLAUR* and *SERPINE1)* of *miR-204* had been observed in cancer tissues as expected since it is known that downregulated expression of miRNAs may upregulate expression of target genes. Expression of the target genes (*DMD* and *CXCL12)* was also downregulated in precancers and cancer tissues probably due to upregulation of *mir3*1 in these tissues. These target genes might have significant roles in progression of diseases since they function in remodeling of extracellular matrix (*MMP9, PLAUR* and *SERPINE1*)^36^, muscle formation and maintenance and tumor suppressor (*DMD*)^37^,^38^,^39^, tumor growth and metastasis (*CXCL12*) 37,38,40, cell shape and motility (WASF3)^41^ and regulation of circadian clock (*KIAA1737*). Except OSMF, expression of only one target gene (*i.e. CXCL12*) of *miR-31* was significantly deregulated in both cancer and two other pre-cancer tissues (Table 4). Lower expression of *CXCL12* was also reported in head and neck cancer due to hypermethylation in promoter region^37^. It has been reported that high expression of *mir31* can immortalize or transform oral keratinocytes^5^ and is also proposed that *mir31* can disrupt DNA repair genes to favor genomic instability and epithelial-mesenchymal transition^4^. Thus, *miR-31* has the potential for malignant transformation of precancer to cancer since upregulated expression of this miRNA has also been observed in leukoplakia and leukoplakia-transformed cancer tissues^42^. So, expression deregulation of *miR-31* and its target gene (*CXCL12)* in pre-cancer and cancer tissues suggest for a common mechanism leading to these diseases. Due to lack of follow-up data in this study, validation of expression in non-progressive and progressive precancer samples could not be performed by us but it was validated by GEO data sets generated from non-progressive and progressive leukoplakia samples. So, expression deregulation of *miR-31* and its target gene (*CXCL12)* seems to have significance in the progression of pre-cancer to cancer, but it needs to be validated in a larger set of samples with follow-up data.

## Methods

### Collection of samples

All experimental protocols were approved by the ethical committee of Indian Statistical Institute, Kolkata. Methods were carried out in accordance with the relevant guidelines and regulations. Written informed consents were taken from all patients and controls. Oral tissue samples were collected with informed consent from five groups of unrelated individuals consisting of four disease groups (oral squamous cell carcinoma, OSCC; leukoplakia, LK; lichen planus, LP and oral submucous fibrosis, OSMF) and one normal/healthy group (Table 5 and Supplementary Table 1A–E) from Pathology Department, Guru Nanak Institute of Dental Science and Research, Kolkata, India. Follow-up of precancer patients could not be performed since they did not turn up to the same hospital for review. So, we do not have data to say how many of our precancers are non-progressive (i.e. not transformed to cancer) or progressive (i.e. transformed to cancer). Tissues were kept in *RNA Later* solution and stored at −80 °C until use. Diseases were confirmed by histopathology from the same department. Though the cancer and pre-cancer patients had tobacco habit but none of the healthy individuals had tobacco habit.

### Isolation of RNA and preparation of cDNA

Total RNA isolation was performed from the tissue samples using Qiagen AllPrep DNA/RNA Mini Kit and RNA yield was quantified by NanoDrop 2000 spectrophotometer (Thermo Scientific, USA). From the RNA samples, random hexamer based cDNA for target genes and miRNA primer specific cDNAs were prepared using Revert Aid First Strand cDNA Synthesis Kit (Thermo Scientific) and Taqman miRNA Reverse Transcription Kit (Applied Biosystems Inc.) respectively.

### Expression assay

#### MiRNA

Expression of 7 miRNAs (hsa-miR-7, hsa-miR-133a, hsa-miR-204, hsa-miR-206, hsa-miR-31, hsa-miR-31*, hsa-miR-1293) were determined from miRNA specific cDNA prepared from RNA samples isolated from oral cancer, three types of pre-cancers and normal tissues. Though 3 miRNAs such as *RNU-44, RNU-48* and *mmu-6*, were primarily included as endogenous controls for normalization of the expression data, only *RNU-44* was finally selected as endogenous control as the expression of the other two miRNAs vary a little from sample to sample. Taqman-based expression assays were performed using miRNA specific probe and primers in 7900HT Fast Real-Time PCR System (Applied Biosystems Inc.)

#### Target genes

Experimentally validated target genes of these 7 miRNAs were obtained from miRTarBase database (release 5.0 and 6.0) (http://mirtarbase.mbc.nctu.edu.tw/) and expression of the targets was, then, checked in another list of expressed genes (i.e. whole transcriptome) in cancer tissues determined by high-through-put RNA-Seq method (unpublished data). Expression of 14 target genes of six miRNAs (hsa-miR-206, hsa-miR-133a, hsa-miR-7, hsa-miR-31, hsa-miR-31* and *hsa-miR-204*) were found to be de-regulated in our RNA-Seq unpublished data. Among these 14 target genes, 10 were selected for expression study in cancer, precancer and normal tissues (Table 4) and expression values of remaining 4 target genes (i.e. *H2AFX, RUNX3, MTHFD1L* and *FYB)* were taken from whole transcriptome data on same cancer tissues (unpublished data). Expression of these 4 target genes could not be determined in precancer tissues due to non-availability of RNA samples. RT-PCR primers were designed for target genes and *Sybr-Green* expression assays were performed in 7900HT Fast Real-Time PCR System. Expression of *RNaseP* gene was used as endogenous control to calculate the expression deregulation of target genes.

Expression data, i.e. *C*_*T*_ value which is the number of cycle at which quantity of PCR product reaches a certain threshold level in log phase of amplification, were converted to normalized expression data i.e. *∆Ct* = *Ct*_expression value of a gene in a tissue_ – *Ct*_expression value of endogenous control gene in the same tissue_. Expression deregulation of a gene in disease tissues compared to control tissues is calculated as mean of *∆∆Ct* values of all samples of a disease group, where mean *∆∆Ct* = mean of *∆Cts* of a gene in disease tissues –mean of *∆Cts* of the same gene in normal tissues. Fold change is calculated as 2^∆∆*Ct*^.

### Statistical analysis

Expression data *(∆Ct)* for each of the five groups were tested for normality (Kolmogorov-Smirnov test). ANOVA and independent t-test were done to check whether the mean *∆∆Ct* of any gene in a disease group is different from that in normal group. The OSMF patients were sub grouped on the basis of presence or absence of dysplasia and checked for any difference in mean *∆Ct*. Benjamini-Hochberg multiple testing corrections were done on the p values to avoid false positive significance.

### Expression validation

For miR-31, expression validation was performed using two data sets of Gene Expression Omnibus (GEO) database^42^. *GSE33299* data set had expression of *miRNAs* from 5 normal oral tissues, 20 leukoplakia and 5 leukoplakia-transformed cancer tissues and GSE62809 data set had miRNA expression profiling from 10 non-progressive and 10 progressive leukoplakias (i.e. turned into oral squamous cell carcinoma within 5 yrs).

## Additional Information

**How to cite this article**: Chattopadhyay, E. *et al*. Expression deregulation of *mir31* and *CXCL12* in two types of oral precancers and cancer: importance in progression of precancer and cancer. *Sci. Rep.* **6**, 32735; doi: 10.1038/srep32735 (2016).

## Supplementary Material

Supplementary Information

## Figures and Tables

**Table 1:** miRNA expression in fold changes in paired and unpaired cancer samples.

MiRNAs	Expression in fold change	
	Paired cancer-normal tissues (n = 18)*	Unpaired cancer-normal tissues (can = 23, nor = 20)*
*hsa-miR-7*	4 ↑	2 ↑
*hsa-miR-31*	5 ↑	30 ↑
hsa-miR-31*	7 ↑	41 ↑
*hsa-miR-1293*	5 ↑	5 ↑
*hsa-miR-133a*	97 ↓	209 ↓
*hsa-miR-204*	27 ↓	23 ↓
*hsa-miR-206*	32 ↓	126 ↓

Paired samples: cancer and adjacent control tissue from same patients (published data)^3^.

Unpaired samples: cancer and control tissues from cancer patients and healthy individuals, respectively.

^*^Significant with P value <0.05 after Benjamini-Hochberg multiple testing correction for all miRNAs.

↑: upregulation; ↓: downregulation of expression.

**Table 2:** Expression of miRNAs in cancer and precancer.

miRNA	Expression in fold change			
	Cancer (n = 23)	Leukoplakia (n = 18)	Lichen planus (n = 12)	Oral submucous fibrosis (n = 23)
*hsa-miR-7*	2 ↑*	6 ↑*	14 ↑*	1
*hsa-miR-31*	30 ↑*	68 ↑*	19 ↑*	3 ↑*
hsa-miR-31*	41 ↑*	65 ↑*	182 ↑*	2 ↑
*hsa-miR-133a*	209 ↓*	20 ↑*	1	3 ↑
*hsa-miR-204*	23 ↓*	4 ↑	3 ↑*	8 ↓*
*hsa-miR-206*	126 ↓*	22 ↑*	3 ↑	2 ↑
*hsa-miR-1293*	5 ↑*	33 ↑*	6 ↑*	1

Expression in fold change of miRNAs in cancer and pre-cancer tissues were calculated with respect to the normal oral tissues from healthy individuals.

^*^Significant after independent t-test with P value < 0.05 and Benjamini-Hochberg multiple testing correction.

↑: upregulation; ↓: downregulation of expression.

**Table 3:** MiRNAs and their target genes selected for expression study.

miRNA	Target genes^b^
*hsa-miR-7*	*TGM2*
*hsa-miR-31*	*DMD, CXCL12, WASF3*
hsa-miR-31*	*KIAA1737*
*hsa-miR-204*	*MMP9, PLAUR, SERPINE1, SNAI2, COL5A3*
*hsa-miR-133a*	*H2AFX, RUNX3, MTHFD1L*
*hsa-miR-206*	*FYB*

Experimentally validated target genes of the miRNAs were selected from miRTarBase (http://mirtarbase.mbc.nctu.edu.tw/) and our unpublished RNAseq data.

^b^Expression of other target genes were not deregulated in our unpublished RNA-Seq expression data, so, was not taken for expression study.

**Table 4:** Fold change in expression of miRNAs and their target genes in cancer and precancer.

miRNA	Cancer (n = 23)	LK (n = 18)	LP (n = 12)	OSMF (n = 23)	Targets	Cancer	LK	LP	OSMF
*hsa-miR-204*	23 ↓*	4 ↑	3 ↑*	8 ↓*	*MMP9*	19 ↑*	2 ↓	3 ↑	1
					*PLAUR*	5 ↑*	2 ↓	3 ↓	2 ↑
					*SERPINE1*	25 ↑*	1	1	3 ↑
					*SNAI2*	3 ↑	2 ↓	2 ↓	1
					*COL5A3*	2 ↑	2 ↓	2 ↓	1
*hsa-miR-31*	30 ↑*	68 ↑*	19 ↑*	3 ↑*	*DMD*	106 ↓*	4 ↓	10 ↓ #	5 ↑
					*CXCL12*	3 ↓*	10 ↓*	17 ↓ #	1
					*WASF3*	2 ↓	3 ↓*	7 ↓ #	1
hsa-miR-31*	41 ↑*	65 ↑*	182 ↑*	2 ↑	*KIAA1737*	2 ↓	2 ↓	4 ↓ #	2 ↑
*has-miR-7*	2 ↑*	6 ↑*	14 ↑*	1	*TGM2*	1	3 ↓	5 ↓	2 ↑
*has-miR-133a*	209 ↓*	20 ↑*	1	3 ↑	*RUNX3*	2 ↑*	ND	ND	ND
					*H2AFX*	1.5 ↑*	ND	ND	ND
					*MTHFDIL*	3 ↑*	ND	ND	ND
*has-miR-206*	126 ↓*	22 ↑*	3 ↑	2 ↑	*FYB*	3 ↑*	ND	ND	ND

Expression fold change of miRNA and target genes in cancer and pre-cancer tissues were calculated with respect to the normal oral tissues from healthy individuals.*Significant, after independent t-test with P value <0.05 and Benjamini-Hochberg multiple testing correction, ↑: upregulation; ↓: downregulation of expression; LK-Leukoplakia, LP-Lichen planus, OSMF-oral sub-mucous fibrosis, ND: not determined due to non-availability of RNA samples.

**Table 5:** Cancer, precancer and normal tissue samples used in this study.

Sample groups	Sample size for	
	miRNA expression	Target gene expression
Cancer	23	23
Leukoplakia	18	16
Lichen planus	12	14
Oral submucous fibrosis^a^	23	15
Normal/healthy	20	15

^a^OSMF samples have 11 dysplasia and 12 without dysplasia tissues.
