# Supplementary material for: Expression deregulation of mir31 and CXCL12 in two types of oral precancers and cancer: importance in progression of precancer and cancer
Source: Sci Rep. 2016 Sep 6;6:32735. doi: 10.1038/srep32735 (PMC5011738; doi:10.1038/srep32735)

**Supplementary Material**

**Title:** Expression deregulation of *mir31*and *CXCL12* in two types of oral precancers and cancer: importance in progression of precancer and cancer

**Authors:**  Esita Chattopadhyay^1^, Richa Singh^1^, Anindita Ray^1^, Roshni Roy^1,2^, Navonil De Sarkar^1,3^ Ranjan Rashmi Paul^4^, Mousumi Pal^4^, Ritesh Aich^5^, and Bidyut Roy^1*^

**Affiliations and Address**^: 1^Human Genetics Unit, Indian Statistical Institute, Kolkata, India

^2^ Present address: Baylor Scott and White Research Institute, Dallas, Texas, USA

^3^ Present address: Fred Hutchinson Cancer Research center, University of Washington, Seattle, USA

^4^Department of Oral Pathology, **Guru Nanak Institute of Dental Science and Research,** 157/F Nilganj Road, Kolkata, 700114, India

^5^Department of Oral Medicine, Dr. R Ahmed Dental College and Hospital, 114- AJC Bose Road, Kolkata 700014, India

*****Corresponding author**:** Bidyut Roy, Human Genetics, Unit, Indian Statistical Institute, 203 BT Road, Kolkata 700108, India. +91-33-25753213, [broy@isical.ac.in](mailto:broy@isical.ac.in)

**Supplementary Figure S1: Normalized expression (i.e. ∆Ct value*) of seven miRNAs in cancer (Can), leukoplakia (LK), lichen planus (LP), oral submucous fibrosis (OSMF) and normal (Nor) groups.**

**
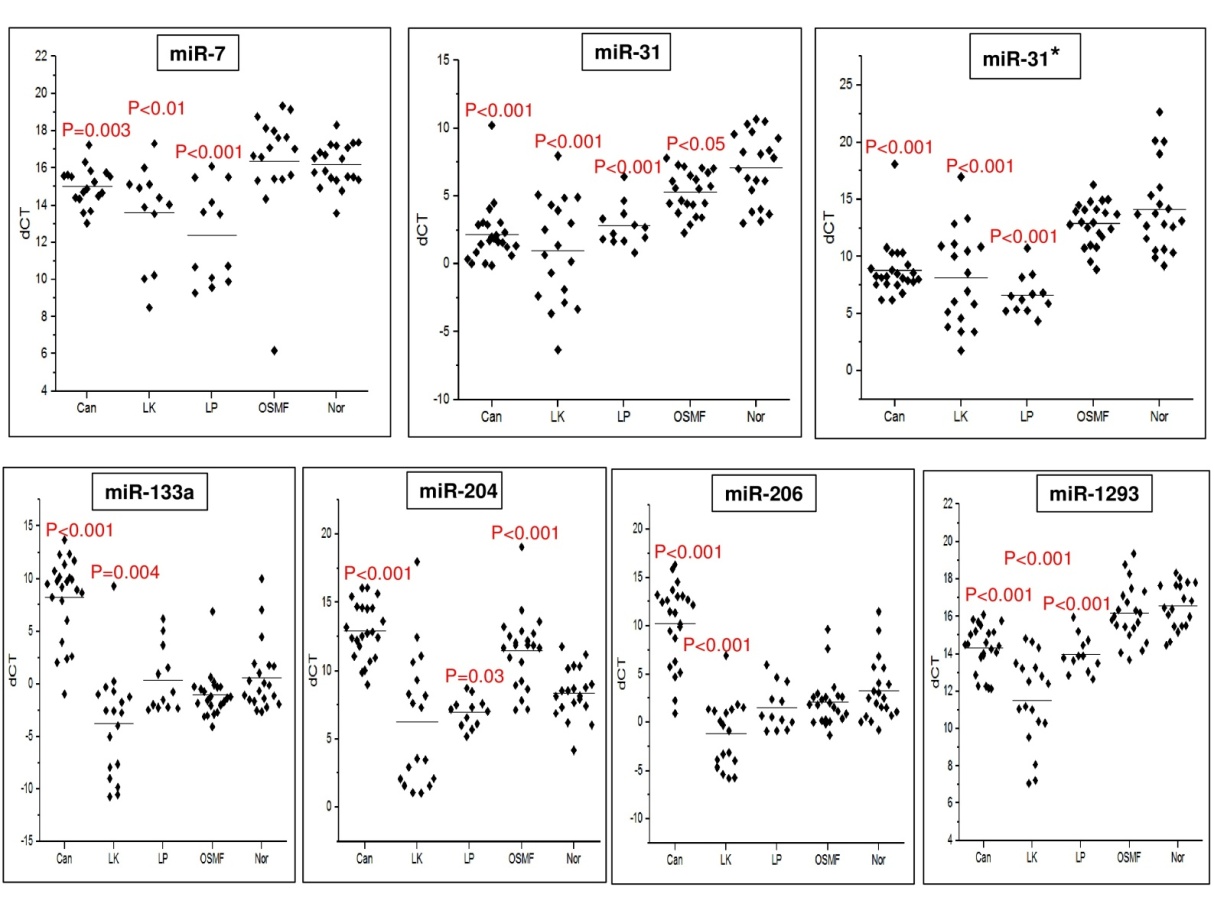
**

***** To get p-values, dCt/ ∆Ct values from cancer and precancers were compared with those from normal tissues. Non-significant p-values were not shown in the figure.

**Supplementary Figure S2: Normalized expression (i.e. ∆Ct value*) of *mir204* and its target genes in cancer (Can), leukoplakia (LK), lichen planus (LP), oral submucous fibrosis (OSMF) and normal (Nor) groups**

**
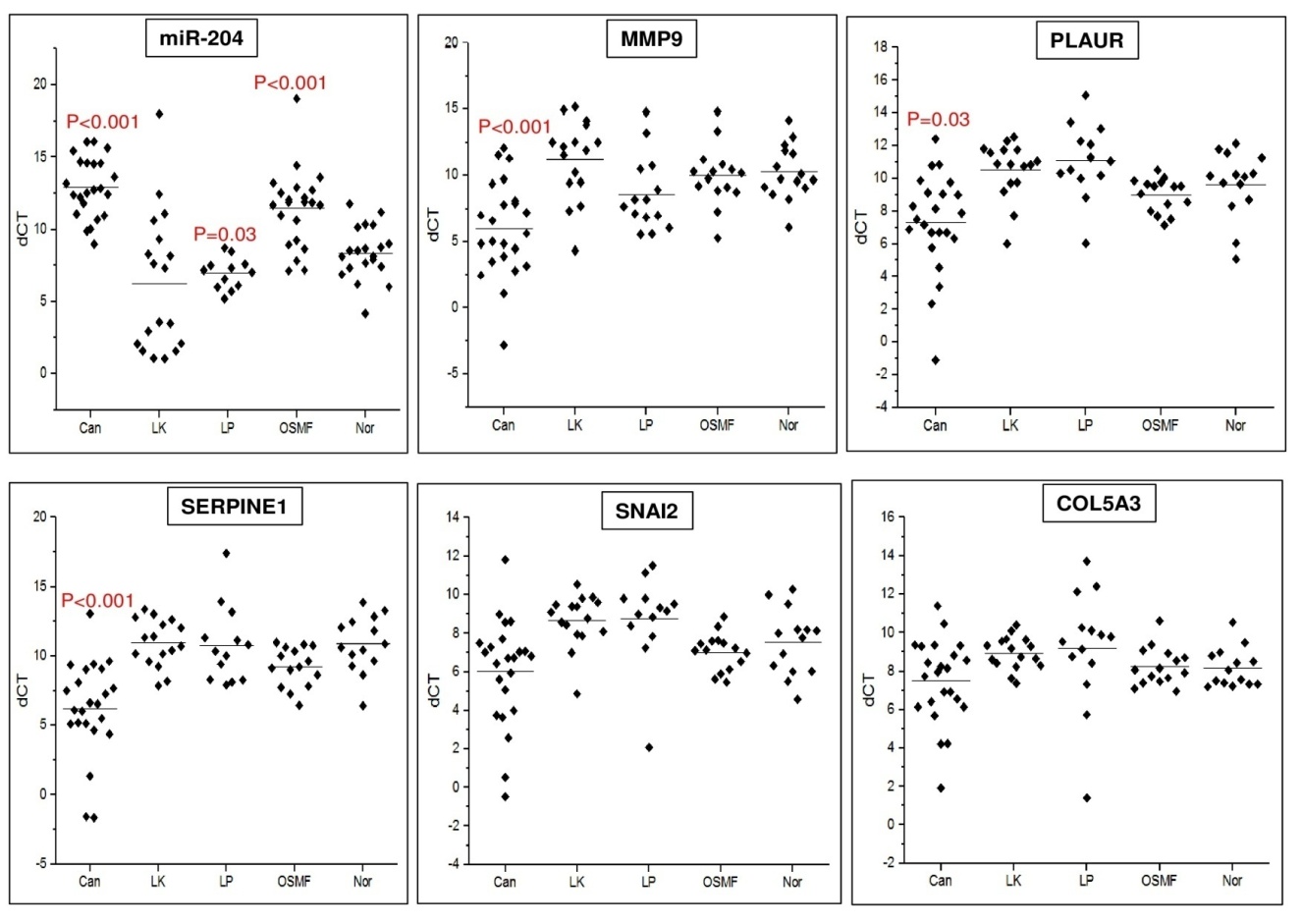
**

***** To get p-values, dCt/ ∆Ct values from cancer and precancers were compared with those from normal tissues. Non-significant p-values were not shown in the figure.

**Supplementary Figure S3: Normalized expression of *mir31* and its target genes in cancer (Can), leukoplakia (LK), lichen planus (LP), oral submucous fibrosis (OSMF) and normal (Nor) groups**

**
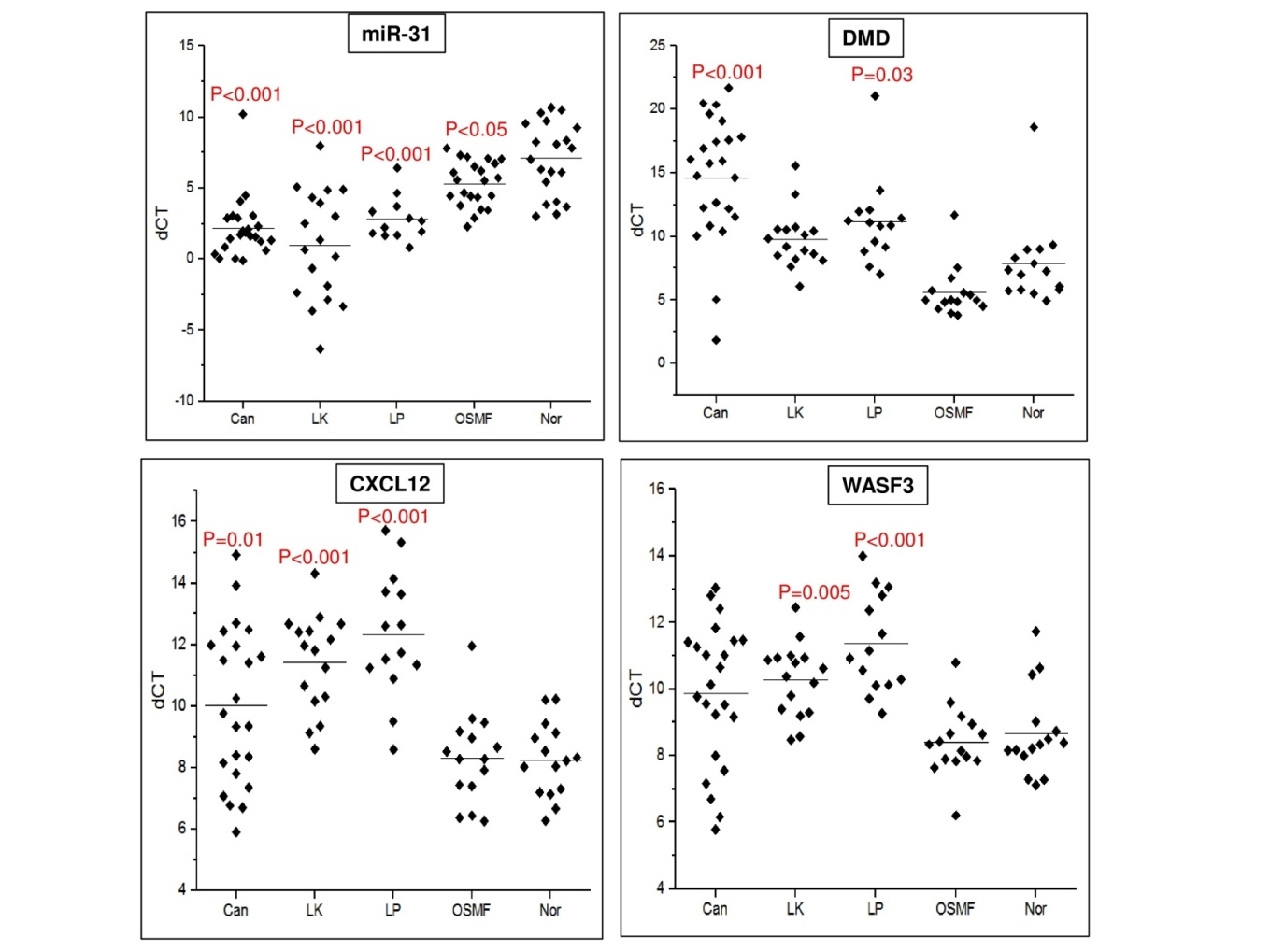
**

To get p-values, dCt/ ∆Ct values from cancer and precancers were compared with those from normal tissues. Non-significant p-values were not shown in the figure.

**Supplementary Figure S4: Normalized expression of *miR-31** and its target gene in cancer (Can), leukoplakia (LK), lichen planus (LP), oral submucous fibrosis (OSMF) and normal (Nor) groups**

**
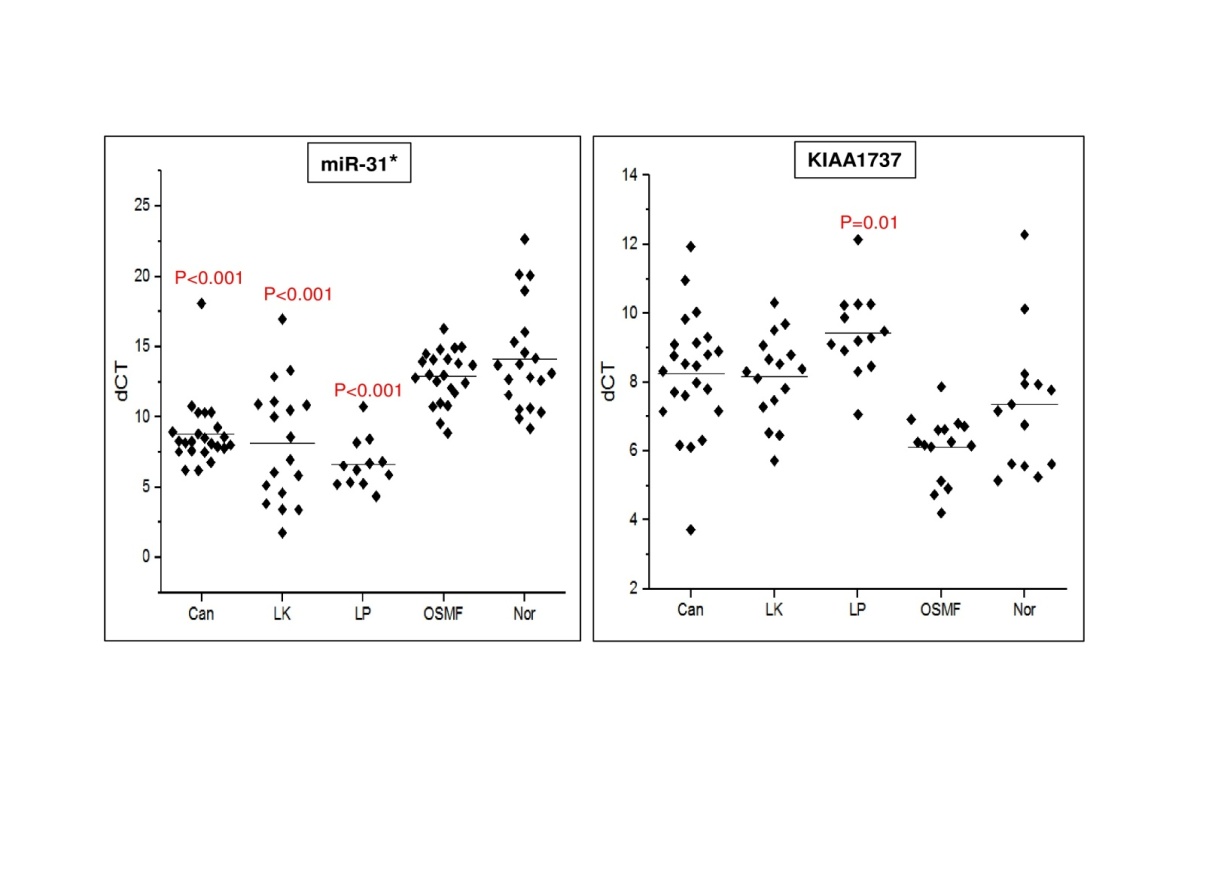
**

To get p-values, dCt/ ∆Ct values from cancer and precancers were compared with those from normal tissues. Non-significant p-values were not shown in the figure.

**Supplementary Figure S5: Normalized expression of *hsa-miR-7* and its target gene *TGM2* in cancer (Can), leukoplakia (LK), lichen planus (LP), oral submucous fibrosis (OSMF) and normal (Nor) groups**

**
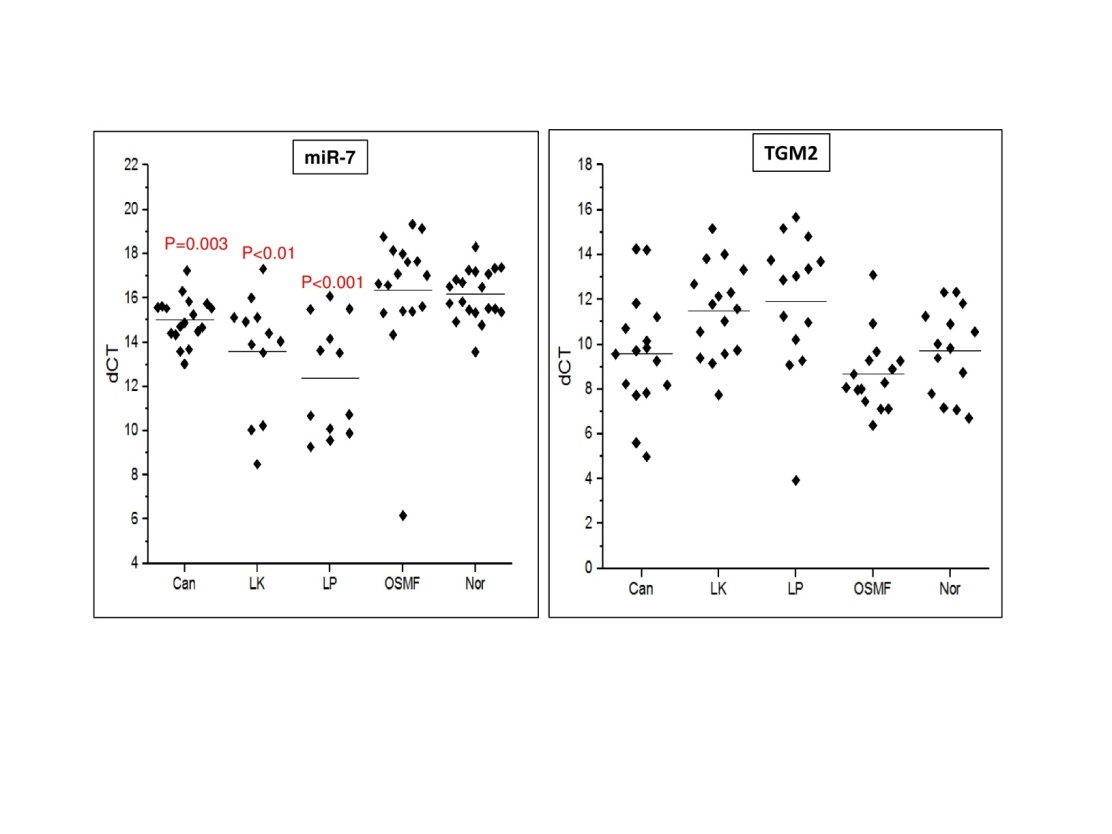
**

To get p-values, dCt/ ∆Ct values from cancer and precancers were compared with those from normal tissues. Non-significant p-values were not shown in the figure.

**Supplementary Table 1A:** Demographic information of oral cancer patients (n=23)

| **Patient Id** | **Sex** | **Age** | **Tobacco habit** | **Doses of tobacco** | | | |
| --- | --- | --- | --- | --- | --- | --- | --- |
|  |  |  |  | **Times/day** | **Years** | **Pack year** | **Chewing year** |
| OSF-25can | M | 63 | Chewing | 6 | 35 | - | 210 |
| NNC1 | F | 50 | Chewing | 3 | 2 | - | 6 |
| NNC2 | M | 71 | Smoking | 3 | 55 | 8.25 | - |
| NNC3 | M | 42 | Chewing | 7 | 12 | - | 84 |
| NNC4 | F | 40 | Chewing | 20 | 16 | - | 320 |
| NNC5 | F | 60 | Chewing | 6 | 40 | - | 240 |
| NNC6 | F | 50 | Chewing | 10 | 30 | - | 300 |
| NNC7 | M | 30 | Smoking  Chewing | 40  10 | 15  15 | 30  - | -  150 |
| NNC8 | F | 62 | Chewing | 4 | 35 | - | 140 |
| NNC9 | F | 57 | No | - | - | - | - |
| NNC10 | M | 45 | Smoking | 10 | 25 | 12.5 | - |
| NNC11 | F | 75 | Chewing | 6 | 25 | - | 150 |
| NNC12 | F | 75 | Chewing | 9 | 50 | - | 450 |
| NNC13 | M | 77 | Chewing | 5 | 35 | - | 175 |
| NNC14 | M | 59 | Smoking  Chewing | 10  3 | 40  40 | 20  - | -  120 |
| NNC15 | F | 39 | Chewing | 4 | 15 | - | 60 |
| AR-1 | M | 55 | Chewing | 7 | 20 | - | 140 |
| AR-2 | M | 50 | Smoking  Chewing | 5  4 | 10  10 | 2.5  - | -  40 |
| AR-3 | M | 55 | Smoking  Chewing | 10  10 | 15  15 | 3.75  - | -  150 |
| AR-4 | M | 36 | Chewing | 8 | 15 | - | 120 |
| AR-5 | F | 62 | No | - | - | - | - |
| CDM-1 | F | 60 | Chewing | 1 | 10 | - | 10 |
| CDM2 | M | 58 | Smoking | 10 | 20 | 10 | - |


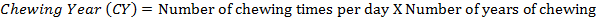

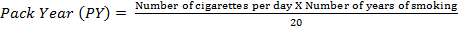

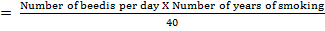


**Supplementary Table 1B:** Demographic information of leukoplakia patients (n=18)

| **Patient Id** | **Sex** | **Age** | **Tobacco habit** | **Doses of tobacco** | | | |
| --- | --- | --- | --- | --- | --- | --- | --- |
|  |  |  |  | **Times/day** | **Years** | **Pack year** | **Chewing year** |
| GNDLK-2 | M | 43 | Smoking  Chewing | 6  9 | 15  15 | 4.5  - | -  135 |
| GNDLK-4 | M | 36 | Smoking | 7 | 15 | 5.25 | - |
| GNDLK-5 | M | 55 | Smoking | 10 | 30 | 15 | - |
| GNDLK-6 | M | 32 | Smoking | 10 | 10 | 5 | - |
| GNDLK-7 | M | 56 | No | - | - | - | - |
| GNDLK-8 | M | 49 | No | - | - | - | - |
| GNDLK-9 | M | 45 | Chewing | 2 | 25 | - | 50 |
| GNDLK-10 | M | 32 | Smoking | 20 | 10 | 10 | - |
| GNDLK-11 | M | 42 | Smoking | 70 | 20 | 70 | - |
| GNDLK-12 | M | 44 | No | - | - | - | - |
| GNDLK-13 | M | 30 | No | - | - | - | - |
| GNDLK-14 | M | 46 | Smoking | 10 | 15 | 3.75 | - |
| GNDLK-15 | M | 61 | Smoking | 15 | 20 | 15 | - |
| GNDLK-16 | M | 48 | Smoking | 20 | 15 | 7.5 | - |
| GNDLK-17 | M | 35 | Smoking | 20 | 20 | 20 | - |
| GNDLK-19 | M | 34 | Smoking | 6 | 10 | 3 | - |
| GNDLK-20 | M | 40 | Smoking | 1 | 7 | 0.35 | - |
| GNDLK-22 | M | 34 | Smoking | 1 | 10 | 0.5 | - |


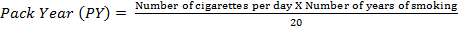

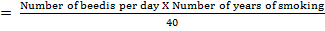

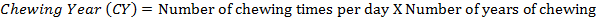


**Supplementary Table 1C:** Demographic information of lichen planus patients (n=14)

| **Patient Id** | **Sex** | **Age** | **Tobacco habit** | **Doses of tobacco** | | | |
| --- | --- | --- | --- | --- | --- | --- | --- |
|  |  |  |  | **Times/day** | **Years** | **Pack year** | **Chewing year** |
| GNDLP-4 | M | 55 | Smoking | 4 | 25 | 2.5 | - |
| GNDLP-6 | M | 41 | Smoking | 4 | 15 | 3 | - |
| GNDLP-9 | F | 37 | No | - | - | - | - |
| GNDLP-10 | M | 15 | No | - | - | - | - |
| GNDLP-11 | F | 45 | No | - | - | - | - |
| GNDLP-12 | F | 52 | No | - | - | - | - |
| GNDLP-15 | F | 46 | No | - | - | - | - |
| GNDLP-18 | F | 35 | Chewing | 6 | 10 | - | 60 |
| GNDLP-20 | M | 48 | Smoking | 10 | 25 | 12.5 | - |
| GNDLP-21 | M | 44 | No | - | - | - | - |
| GNDLP-25 | F | 45 | No | - | - | - | - |
| GNDLP-26 | F | 31 | No | - | - | - | - |
| GNDLP-31 | M | 55 | Smoking | 10 | 20 | 10 | - |
| GNDLP-32 | F | 33 | No | - | - | - | - |


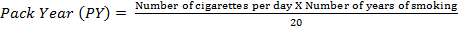

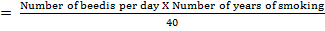

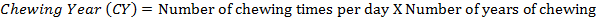


**Supplementary Table 1D:** Demographic information of oral submucous fibrosis patients (n=23)

| **Patient Id** | **Sex** | **Age** | **Tobacco habit** | **Doses of tobacco** | | | |
| --- | --- | --- | --- | --- | --- | --- | --- |
|  |  |  |  | **Times/day** | **Years** | **Pack year** | **Chewing year** |
| OSF-1 | F | 28 | Chewing | 4 | 6 | - | 24 |
| OSF-2 | F | 56 | Chewing | 4 | 30 | - | 120 |
| OSF-3 | M | 29 | Chewing | 15 | 15 | - | 225 |
| OSF-4 | M | 22 | Smoking | 3 | 3 | 0.45 | - |
| OSF-5 | F | 60 | Chewing | 10 | 30 | - | 300 |
| OSF-6 | F | 65 | Chewing | 5 | 50 | - | 250 |
| OSF-7 | M | 25 | Smoking  Chewing | 2  7 | 6  8 | 0.6  - | -  56 |
| OSF-8 | F | 52 | Chewing | 5 | 15 | - | 75 |
| OSF-11 | M | 36 | Chewing | 10 | 20 | - | 200 |
| OSF-12 | F | 43 | Chewing | 3 | 10 | - | 30 |
| OSF-13 | M | 24 | Chewing | 6 | 0.5 | - | 3 |
| OSF-14 | F | 45 | Chewing | 5 | 20 | - | 100 |
| OSF-15 | M | 25 | Chewing | 6 | 3 | - | 18 |
| OSF-16 | M | 32 | Chewing | 5 | 10 | - | 50 |
| OSF-17 | M | 25 | Chewing | 5 | 8 | - | 40 |
| OSF-19 | M | 28 | Smoking | 8 | 8 | 3.2 | - |
| OSF-20 | M | 22 | Chewing | 6 | 4 | - | 24 |
| OSF-21 | M | 34 | Smoking  Chewing | 5  50 | 15  15 | 3.75  - | -  750 |
| OSF-22 | F | 38 | Chewing | 7 | 10 | - | 70 |
| OSF-23 | F | 32 | Chewing | 4 | 7 | - | 28 |
| OSF-24 | M | 30 | Smoking | 6 | 8 | 2.4 | - |
| OSF-26 | M | 21 | Smoking  Chewing | 10  1 | 3  3 | 1.5  - | -  3 |
| NNC15-OSF | F | 39 | Chewing | 4 | 15 | - | 60 |


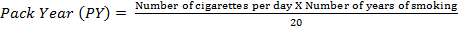

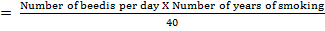

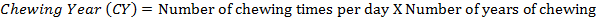


**Supplementary Table 1E:** Demographic information of healthy normal individuals (n=20)

| **Patient Id** | **Sex** | **Age** | **Tobacco habit** |
| --- | --- | --- | --- |
|  |  |  |  |
| N2 | F | 61 | No |
| N3 | M | 32 | No |
| N4 | M | 25 | No |
| N5 | F | 65 | No |
| N6 | F | 32 | No |
| N7 | M | 39 | No |
| N9 | M | 47 | No |
| 13N | M | 24 | No |
| N10 | M | 64 | No |
| N11 | M | 42 | No |
| N12 | F | 30 | No |
| N13 | M | 49 | No |
| N14 | M | 37 | No |
| N15 | M | 16 | No |
| N16 | F | 27 | No |
| N17 | M | 62 | No |
| N18 | M | 55 | No |
| N19 | M | 40 | No |
| N20 | M | 25 | No |
| N21 | M | 41 | No |


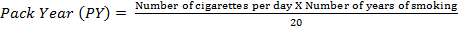

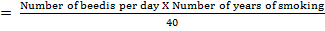

Supplement: Supplementary Information [file srep32735-s1.docx]
